# Technology usage for teaching and learning in nursing education: An integrative review

**DOI:** 10.4102/curationis.v45i1.2261

**Published:** 2022-06-15

**Authors:** Gopolang Gause, Isaac O. Mokgaola, Mahlasela A. Rakhudu

**Affiliations:** 1 School of Nursing, Faculty of Health Sciences, North-West University, Mmabatho, South Africa

**Keywords:** COVID-19, digital learning, online learning, nursing, teaching and learning, technology use

## Abstract

**Background:**

The increasing availability of technology devices or portable digital assistant devices continues to change the teaching-learning landscape, including technology-supported learning. Portable digital assistants and technology usage have become an integral part of teaching and learning nowadays. Cloud computing, which includes YouTube, Google Apps, Dropbox and Twitter, has become the reality of today’s teaching and learning and has noticeably improved higher education, including nursing education.

**Objectives:**

The aim of this integrative literature review was to explore and describe technology usage for teaching and learning in nursing education.

**Method:**

A five-step integrative review framework by Whittemore and Knafl was used to attain the objective of this study. The authors searched for both empirical and non-empirical articles from EBSCOhost (health information source and health science), ScienceDirect and African Journals Online Library databases to establish what is already known about the keywords. Key terms included in literature search were *coronavirus disease 2019* (*COVID-19*), *digital learning, online learning, nursing, teaching and learning, and technology use.*

**Results:**

Nineteen articles were selected for analysis. The themes that emerged from this review were (1) technology use in nursing education, (2) the manner in which technology is used in nursing education, (3) antecedents for technology use in nursing education, (4) advantages of technology use in nursing education, (5) disadvantages of technology use in nursing education and (6) technology use in nursing education amidst COVID-19.

**Conclusion:**

Technology in nursing education is used in both clinical and classroom teaching to complement learning. However, there is still a gap in its acceptance despite its upward trend.

**Contribution:**

The findings of this study contribute to the body of knowledge on the phenomenon of technology use for teaching and learning in nursing education.

## Introduction

Technology devices, such as smartphones, have become the first and the last thing that human beings interact with on a daily basis (Alsayed, Bano & Alnajjar 2020:244). Consequently, the authors add that this has created a strong bond between human beings and their technology devices (Alsayed et al. 2020:244). The increasing availability of technology devices or portable digital assistant (PDA) devices continues to change the teaching-learning landscape, including but not limited to technology-supported learning (Forehand, Miller & Carter 2017:51). The use of technology makes learning fun and exciting, and it cuts across all disciplines including nursing education (Forehand et al. 2017:51). Technology usage in teaching and learning has risen to its peak recently given the current coronavirus disease 2019 (COVID-19) pandemic and its social distancing protocols. Higher education institutions including nursing education institutions globally have resorted to online learning to continue teaching and learning amidst COVID-19 pandemic.

Technology usage plays a vital role in the facilitation of learning in higher education institutions (Al-Hariri & Al-Hattami 2017:84). Portable digital assistants and technology usage have become an integral part of contemporary teaching and learning (Hashim 2018:2). The authors add that cloud computing, which includes YouTube, Google Apps, Dropbox and Twitter, has become the reality of today’s teaching and learning and has noticeably improved higher education, including nursing education. Similar to the context of this review, technology seems to be the backbone of teaching and learning wherein information searching, teaching and learning, and assessment are all reliant on technology. Students mainly use technology and technology devices for web browsing and class preparation and to document and record class proceedings for referral (Alsayed et al. 2020:243). Several studies have highlighted the benefits of technology usage in nursing education that is associated with its exponential growth (MacKay, Anderson & Harding 2017:3; Márquez-Hernández et al. 2020:5; Subedi et al. 2020:73).

According to Subedi et al. (2020:73), technology use in teaching and learning is flexible, minimises travelling and thus is cost-effective, and it allows family time as classes can be attended at the comfort of one’s home. However, some perceive the home environment as disruptive and less conducive for teaching and learning. The benefits of technology usage in teaching and learning are not only limited to classroom setting or theoretical aspect of nursing education. In a study conducted by Mackay Anderson and Harding (2017:3) and Márquez-Hernández et al. (2020:5), students reported that technology and PDAs allow them to have access to a range of websites that enables them to make on-the-spot and sound clinical decisions when placed at clinical facilities for work-integrated learning (WIL). Furthermore, integration of technology usage with clinical settings has proven to be the driver of innovative ideas in making sound clinical decisions (DiMattio & Hudacek 2020:4). Despite the notable benefits of technology use for teaching and learning in nursing education, there are still challenges that face this fast-growing modality of teaching and learning.

There are still nurse managers and educators who feel that technology usage in nursing education is disruptive, especially when used at clinical settings (Alsayed et al. 2020:244). Some nurse educators prefer traditional teaching and learning than technology usage in teaching and learning (Al-Hariri & Al-Hattami 2017:84). In addition, hardware and software issues, connectivity, security and safety of personal information, and lack of face-to-face interaction are some other challenges facing optimum use of technology in teaching and learning (Dhir et al. 2017:875). Furthermore, non-proficiency regarding the use of Information and Communication Technology (ICT) and PDAs is also a challenge for technology usage in nursing education and also a factor contributing to its counterproductivity (Forehand et al. 2017:51). This is seen mainly in students with little or no exposure to the use of computer during their basic schooling.

## Aim

The aim of this integrative literature review was to explore and describe technology usage for teaching and learning in nursing education.

### Integrative review question

What evidence exists on technology usage for teaching and learning in nursing education?

## Design and methods

**FIGURE 1:**
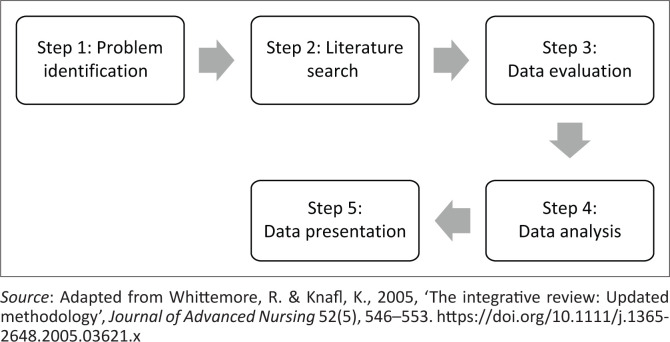
Integrative review framework.

This study followed an integrative literature review design to explore and describe the existing evidence on technology usage for teaching and learning in nursing education. Integrative literature review design is described as a review method that aims to summarise theoretical or empirical evidence to generate a new understanding of a specific phenomenon or a healthcare problem (Broome as quoted in Whittemore & Knafl 2005:546). As Chalmers et al. (2014:156) put it, integrative reviews are important in ensuring that new primary research is generated from the full knowledge of the existing literature. Similar to this present review, researchers intend to generate new knowledge from the existing literature on technology usage for teaching and learning in nursing education. A five-step integrative literature review framework by Whittemore and Knafl (2005:549) was used to attain the aim of this review. The steps are presented in Figure 1.

### Step 1: Problem identification

Given the above background it is clear that a substantial amount of literature exists on the phenomenon in question. However, many of the studies conducted in subjects related to the use of technology in nursing education were conducted prior to COVID-19 pandemic, which necessitated the need for technology use in higher education including nursing education. Given the current impact of COVID-19 pandemic on teaching and learning in undergraduate nursing education, integrative literature review is mandatory in order to help nurse educators and researchers keep up-to-date with a large and rapidly growing body of evidence on technology use for teaching and learning in nursing education.

The purpose of this review was to explore and describe the evidence that exists on literature regarding technology usage for teaching and learning in nursing education. The variables in this review are technology usage and teaching and learning, and the population comprised articles retrieved from the initial search using the literature search strategy that is explained below.

### Step 2: Literature search

**FIGURE 2:**
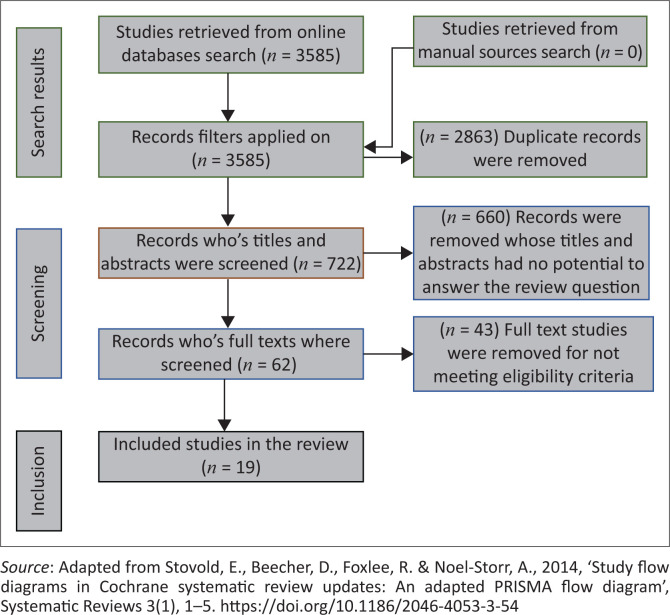
PRISMA flow diagram.

The authors searched for both empirical and non-empirical articles from EBSCOhost (health information source and health science), ScienceDirect and African journals. According to Whittemore and Knafl (2005:549), online databases have proved to be effective and efficient when coming to literature search stage of integrative review. The authors purposely chose the aforementioned online databases because of their potential to answer the review question as they publish health science-related articles. In addition, literature search was conducted only on electronic databases given the current risk of COVID-19 infection from contact with objects and surfaces. A Boolean search method was used to search for literature in the aforementioned databases using the following keywords: *technology use, digital learning, online learning, nursing, and teaching and learning*. Connecting words such as ‘and’ and ‘or’ were used to search for literature. For example, literature search was conducted as ‘“Technology use” or “Digital learning” or “Online learning”’. In order to enhance the literature search, synonyms such as e-learning for online learning and remote learning for digital learning were used to broaden the search of literature. The time frame was between 2016 and 2021. The rationale for including literature from 2016 was the fact that technology use has long been in the shores of nursing education. However, the authors acknowledge that the review was conducted in the COVID-19 era where technology use was at its peak. Therefore, the authors intended to explore and describe the evidence that exists on technology use in nursing education, without narrowing it to COVID-19, although acknowledging its impact on teaching-learning landscape. Specificity of literature search was ensured by the use of keywords that made the search specific to the aim of this integrative review. The use of synonyms and time frame ensured a comprehensive literature search. For each database, the authors applied filters such as ‘English’ and ‘full texts’ only to the retrieved articles in order to ensure that articles with a potential to answer the review question are subjected to screening process. An adapted PRISMA flow diagram from Stovold et al. (2014:3) was then used to further screen the studies for their relevance and potential inclusion in this present review (Figure 2). To ensure rigour, this integrative review was conducted under the supervision of experienced researchers.

**FIGURE 3:**
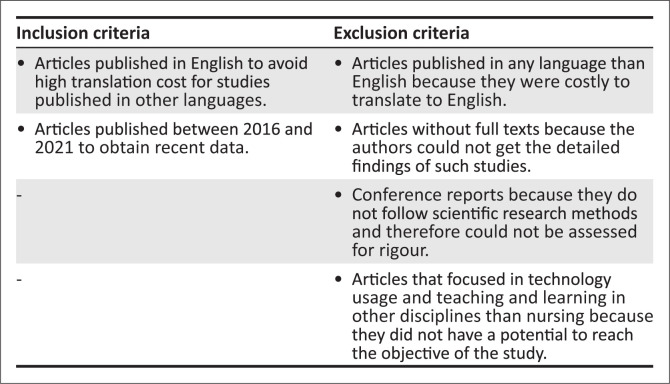
Inclusion and exclusion criteria.

Furthermore, Whittemore and Knafl (2005:549) emphasise that sampling decisions such as keywords and search terms and inclusion and exclusion criteria must be rationalised and documented. Therefore, the rationale for the inclusion and exclusion criteria is described in Figure 3.

### Step 3: Data evaluation

Nineteen articles were finally selected for inclusion in this review. The authors adapted the quality appraisal criteria by Kangasniemi, Pakkanen and Korhonen (2015:1748) to appraise the quality of studies. Studies were appraised on four quality domains, namely, aims and objectives, study design, research methods and limitations. Studies were further evaluated on a three-point scale and were classed as ‘yes’, ‘poor’ or ‘not reported’ presented as ‘Y’, ‘P’ or ‘NR’, respectively (Table 1). The choice of this quality appraisal tool was based on its track record as it was used by Kangasniemi et al. (2015:1748) and later adapted by Moagi et al. (2021:4) in their study, thus making the tool valid and reliable. However, articles were not excluded based on data evaluation. Most articles that were included in this present review followed quantitative approach wherein the researchers used surveys and questionnaires for data collection. This was followed by qualitative approach with interviews as their main method of data collection. The remaining articles followed mixed methods approach.

**TABLE 1:** Quality appraisal of articles.

No.	Author(s) year, Country	Purpose	Design	Highlights of key findings	Quality appraisal criteria (Scale: Y = yes, P = poor, NR = not reported
1.	Barisone et al. (2019), Italy	To explore the perception and effectiveness of web-based learning in facilitating the development of clinical skills in undergraduate nursing students.	Qualitative descriptive study	Clinical learning is fundamental to prevent and know how to manage risky situations.	(Y) Aims and objectives clearly described. (Y) Study design adequately described. (Y) Research methods appropriate. (Y) Limitations presented.
				Therefore, it is important to facilitate the learning of gestural skills and can be effectively done with the support of technology.	
				These learning instruments, which are easy to use and access, could reinforce the knowledge development process by acting as a bridge between theory and practice.	
2.	Chang and Lai (2021)	To understand the experience of nursing students in using virtual reality skill learning process.	Qualitative exploratory descriptive design	Most students expressed that because the virtual reality environment was responsive to hand touch, the gestures were easier to learn.	(P) Aims and objectives clearly described. (Y) Study design adequately described. (Y) Research methods appropriate. (NR) Limitations presented.
				By enabling learners who are unfamiliar with the technology to understand the comprehensive process, the system can assist in the learning process.	
				Furthermore, the system provides learning resources on demand, thus creating an independent and stress-free learning environment.	
3.	Coopasami Knight and Pete (2017), South Africa	To assess the students’ readiness to make a shift from traditional learning to the technological culture of e-learning at a university in Durban.	Quasi-experimental interrupted time series	Less than half (47%) initially knew what e-learning was and this improved to 75% post-intervention.	(P) Aims and objectives clearly described. (Y) Study design adequately described. (Y) Research methods appropriate. (Y) Limitations presented.
				Just less than half (46%) of the participants thought that e-learning could lead to social isolation, but most DUT nursing students live in residence and enjoy an active social life.	
				Turning to the overall e-learning readiness score, most (72%) of the participants were categorised as ‘proceed with caution’.	
4.	Foronda et al. (2016), United States of America	Evaluation of vSIM for Nursing: A Trial of Innovation.	Descriptive, mixed-methods design	Respondents reported that the content of the virtual simulation was directly relevant to their role as a nurse (61% strongly agree and 39% agree).	(Y) Aims and objectives clearly described. (Y) Study design adequately described. (Y) Research methods appropriate. (Y) Limitations presented.
				Nearly, all the nursing students who participated recommended vSIM for future use, suggesting that the virtual simulation experience was a positive one.	
5.	Harerimana and Mtshali (2019), South Africa	To explore nursing students’ perceptions and expectations regarding the use of technology in nursing education.	Cross-sectional descriptive quantitative research	The students perceived that technology was used by educators to deliver instructions (3.77 ± 1.19), to maintain students’ attention (3.77 ± 1.19) and to make connections to the learning process through audio or video material (3.64 ± 1.36).	(Y) Aims and objectives clearly described. (Y) Study design adequately described. (Y) Research methods appropriate. (NR) Limitations presented.
				Nursing students reported that educators use technology for various academic purposes.	
				Overall, the majority of the nursing students had high expectations for nurse educators to use Moodle (88.7%), search tools (75.3%), published electronic resources (70.7%) and early-alert systems designed to catch potential academic trouble as soon as possible (70.0%).	
6.	Maboe (2017), South Africa	To determine how the discussion forum as an online interactive tool be used in an ODL institution to enhance student-to-student and student-to-lecture online interactions.	Quantitative descriptive study	Computers and cell phones with Internet access allow students access to the discussion forum on the website of an ODL institution.	(Y) Aims and objectives clearly described. (Y) Study design adequately described. (Y) Research methods appropriate. (Y) Limitations presented.
				Thirty-four (39.5%) of the respondents agreed that the online discussion forum allows them to study with their peers; 28 (32.5%) strongly agreed; and 17 (19.8%) were unsure.	
				About 20.0% of the respondents indicated that they get no support from lecturers and fellow students when they interact online.	
7.	Mackay et al. (2017), New Zeeland	To describe the process of introducing teaching innovation and to explore clinical lecture perceptions and experience of the use of mobile smart devices to support student learning.	Qualitative descriptive study	The use of the iPad enabled a rich range of resources to be available to both the lecturer and the student.	(Y) Aims and objectives clearly described. (Y) Study design adequately described. (Y) Research methods appropriate. (NR) Limitations presented.
				The lecturers were very positive about the immediate and portable connectivity to a rich range of resources.	
				There were reports that it enhanced the students’ critical thinking.	
8.	Mawere, Mukonza and Kugara (2021), South Africa	The paper explores the experiences faced by first entering students from rural-based institutions on the use of digital learning during the coronavirus pandemic in South Africa.	Participatory action research method	One of the critical findings in this paper is that most rural institutions are not ready or rather lack capacity to cater for disadvantaged students.	(Y) Aims and objectives clearly described. (Y) Study design adequately described. (Y) Research methods appropriate. (Y) Limitations presented.
				The other significant finding was the absence of devices to connect for digital learning.	
9.	O’Connor and Andrews (2018)	To understand the perspectives of nursing students in relation to using smartphones and mobile apps to enhance learning in clinical environments.	Quantitative cross-sectional descriptive design	Of note there was an upward trend in those who used educational apps in practice.	(Y) Aims and objectives clearly described. (Y) Study design adequately described. (P) Research methods appropriate. (Y) Limitations presented.
				Although many students did not actively use apps to help them learn clinical settings, when asked if they would consider doing so, the majority said ‘yes’.	
				Compared to other approaches, students ranked mobile apps as the third most useful source of information for learning in practice.	
10.	O’Connor and LaRue (2021), United Kingdom	To describe how health informatics is being integrated into a Bachelor of Nursing programme in the United Kingdom.	Case study	A wider evaluation of the new nursing informatics curricula and pedagogic framework is currently underway to determine its usefulness in giving nursing students the competencies they need to become skilled in digital health.	(P) Aims and objectives clearly described. (P) Study design adequately described. (Y) Research methods appropriate. (NR) Limitations presented.
11.	Oducado and Soriano (2021), Philippines	To examine nursing students’ attitudes towards e-learning in two selected nursing schools in the Philippines.	Descriptive, cross-sectional research design	This is an expected finding during this uncertain and unprecedented time of crisis and as the educational system transition from the traditional delivery of instruction to a more flexible yet unpopular modality of teaching and learning.	(Y) Aims and objectives clearly described. (Y) Study design adequately described. (Y) Research methods appropriate. (Y) Limitations presented.
				The nursing students may be unfamiliar with and not fully prepared for the new modality in learning.	
12.	Singh et al. (2021), India	To provide preliminary data to the stakeholders regarding the feasibility and acceptability of e-learning.	Quantitative online survey	Computer and Internet usage and availability of dedicated space at home (where there are no environmental distractions) to attend online classes determine the feasibility or practicability of e-learning.	(P) Aims and objectives clearly described. (P) Study design adequately described. (Y) Research methods appropriate. (NR) Limitations presented.
				Seamless Internet connectivity is of paramount importance to attend classes without interruption.	
				Network-related issues were frequently reported by a significant percentage of students.	
13.	Suliman et al. (2021)	To investigate the experiences of undergraduate nursing students during their first uses of OL to increase the understanding of their encountered opportunities and challenges.	Qualitative exploratory descriptive design	A combination of the following platforms was used to facilitate the OL of the participants: E-learning (12 students), Microsoft Team (seven students), Zoom (16 students), Skype (seven students), WhatsApp (15 students), YouTube (eight students) and Facebook (11 students).	(Y) Aims and objectives clearly described. (Y) Study design adequately described. (Y) Research methods appropriate. (NR) Limitations presented.
				Most study participants reported having poor skills in using OL technology.	
				Half of the students reported that they had episodes of Internet failure whilst attending classes, taking quizzes or submitting assignments, which provoked feelings of helplessness and contributed to their dissatisfaction with the online experience.	
14.	Toothaker (2018), Pennsylvania	To assess the millennial perceptions and attitudes of clickers on learning during traditional lecture series.	Mixed-method design	The qualitative results reflect positive perception of millennial nursing students’ use of clickers.	(Y) Aims and objectives clearly described. (Y) Study design adequately described. (Y) Research methods appropriate. (NR) Limitations presented.
				Ninety-one percent of the students agreed or strongly agreed that the use of clickers helped them to develop a better understanding of the subject matter when compared to traditional lecture-based classes.	
				Eighty-nine per cent of the nursing students felt that the clicker question provided the professors to respond to concepts not understood in the classroom.	
15.	Uprichard (2020), Manchester	To explore both the benefits of and barriers to e-learning.	Qualitative exploratory descriptive study	One of the clear benefits of using e-learning to deliver training is the flexibility of the location and time it needs to be completed.	(P) Aims and objectives clearly described. (P) Study design adequately described. (P) Research methods appropriate. (NR) Limitations presented.
				One of the main barriers to the use of e-learning is technical difficulty.	
				Another barrier to the use of e-learning is technological illiteracy.	
16.	Van Vuuren, Goon and Seekoe (2018), South Africa	The perceptions of nurse educators regarding the use of high fidelity simulation in nursing education.	Quantitative descriptive research design	Low and medium fidelity simulators are widely utilised in both classroom and clinical settings.	-
				The fact that most participants agreed that high fidelity simulators help to reduce errors and improve teaching shows that patient safety is also a priority.	
17.	Verkuyl and Mastrilli (2017), Canada	Virtual Simulations in Nursing Education: A Scoping Review.	Arksey and O’Malley’s scoping methodology	The participants in the review in general exhibited enthusiasm for virtual simulation as a teaching strategy in nursing.	-
				These findings are encouraging to faculty members who are exploring innovation in technology and provide support for further development and implementation of virtual simulations.	
18.	Willemse Jooste, and Bozalek (2019), South Africa	To explore the experiences of undergraduate nursing students who participated in an authentic mobile learning enactment aimed at enhancing their learning experiences.	Qualitative contextual design	Challenges experienced with data/airtime/Wi-Fi, impaired communication because of poor network access and use of mobile devices in practice perceived as unprofessional.	(Y) Aims and objectives clearly described. (Y) Study design adequately described. (Y) Research methods appropriate. (NR) Limitations presented.
19.	Zarandona et al. (2019), Spain	To characterise the use of smartphones by nursing students and to assess their opinions about the use of such phones as a distracting factor during clinical practicum and smartphone restriction policies.	Quantitative cross-sectional descriptive study	Overall, 23.3% of participants admitted to having used their smartphone for personal reasons at least once during their practicum.	(Y) Aims and objectives clearly described. (P) Study design adequately described. (Y) Research methods appropriate. (Y) Limitations presented.
				Most students (98.3%) used their smartphones for accessing social networks, followed by university resources (42.3%).	
				Other cited uses were as a tool for communication and coordination with other team members (19.4%) and for accessing apps to support patient care (13.4%).	

*Source*: Adapted from Kangasniemi, M., Pakkanen, P. & Korhonen, A., 2015, ‘Professional ethics in nursing: An integrative review’, *Journal of Advanced Nursing* 71(8), 1744–1757. https://doi.org/10.1111/jan.12619

vSIM, virtual simulation; ODL, open distance learning; DUT, Durban University of Technology; OL, online learning.

### Step 4: Data analysis

Articles were analysed independently by the authors following six steps of thematic analysis, namely, familiarisation, coding, generating themes, reviewing themes, defining and naming themes, and writing up. Firstly, the authors went through included articles before analysis. Secondly, the authors highlighted phrases of the texts which described the content of such texts. Thirdly, themes were then generated from the pattern of the codes which were identified in the literature. Fourthly, the authors checked the accuracy of the themes to ensure that they are a true representation of the included studies. Fifthly, the authors labelled each theme, and this was succeeded by the sixth step which is writing up. An inductive approach of thematic analysis was used. This implies that the authors allowed data to generate themes as explained earlier. As six themes emerged: (1) technology use in nursing education, (2) the manner in which technology is used in nursing education, (3) antecedents for technology use in nursing education, (4) advantages of technology use in nursing education, (5) disadvantages of technology use in nursing education and (6) technology use in nursing education amidst COVID-19.

### Ethical considerations

Ethical clearance to conduct this study was obtained from the North-West University Health Research Ethics Committee (NWU-HREC) (number: NWU-02071-20-A1). Ethical standards for conducting the research were followed in this article, even though it was a review and not conducted with human, plant or animal participants. Studies that had ethical approval were included.

### Step 5: Data presentation

In this section, the authors outline and discuss the results of this integrative review.

## Results

The key results of this integrative review are described in this section under the following themes: (1) technology use in nursing education, (2) the manner in which technology is used in nursing education, (3) antecedents for technology use in nursing education, (4) advantages of technology use in nursing education, (5) disadvantages of technology use in nursing education and (6) technology use in nursing education amidst COVID-19. The results of this study included surrogate terms for technology usage for teaching and learning, such as ‘online learning’, ‘technology use in teaching and learning’, ‘e-learning’ and ‘virtual learning’.

### Theme 1: Technology use in nursing education

The first theme discusses the frequency of technology use for teaching and learning in both classroom and clinical nursing education.

Information and communication literacy, information systems management and citizen digital health literacy are amongst the individual descriptors of learning that students should achieve in each of the key areas (O’Connor & LaRue 2021:3). This implies that digital literacy is amongst the critical cross-fields for every graduate. As a result, the increase in the number of people who use computerised devices such as smartphones is relatively proportional to the amount of time spent on these devices (Zarandona et al. 2019:70). For the students belonging to the millennial generation, technology devices have become the important tool for interaction (Willemse et al. 2019:72). This is no exception in nursing education wherein the use of technology devices both in the class and in clinical placements is an order of the day. The use of mobile applications (apps) in nursing education has been ranked top in comparison to peer learning and clinical placement coordinators (O’Connor & Andrews 2018:174). The authors further highlighted that an upward trend in technology use for teaching and learning continues to be noticed, especially in clinical nursing education.

### Theme 2: Manner in which technology is used in nursing education

This theme discusses the manner in which technology is used for teaching and learning both in clinical and classroom settings.

#### Manner in which technology is used in classroom nursing education

According to Harerimana and Mtshali (2019:6), technology in nursing education is primarily used for communication of instructions to the students to enhance their creativity and critical thinking skills and also for building relationships with stakeholders in nursing education. The authors add that technology in nursing education is used to maintain student attention in the classroom and to corroborate theoretical learning through the use of audio virtual aids. Teaching and learning in classroom is achieved through the use of mediums such as Microsoft Teams, Skype and Zoom together with a range of available social media platforms. Using those mediums, teaching and learning is then facilitated either synchronously or asynchronously through the use of PowerPoint presentations, didactic lectures, video-based learning, case-based learning, prerecorded lectures, quizzes or online whiteboards (Singh et al. 2021:4). Moreover, technology in classroom nursing education can also be used to manage academic dishonesty through the use of software such as Turnitin (Harerimana & Mtshali 2019:9).

#### Manner in which technology is used in clinical nursing education

Technology use in clinical teaching can be split into two entities which are interlinked, namely, the clinical placement for WIL and simulation lab. Generally, there has been a notable increase in the use of technology applications for teaching and learning in clinical nursing education of late (O’Connor & Andrews 2018:174). According to O’Connor and Andrews (2018:174) and Zarandona et al. (2019:69), the most common uses of technology at the clinical placement for WIL include the use of software to access applications such as calculators, drug reference guide, disease and disorder books, and medical dictionary. This allows students to get the comfort of accessing instant references in their pocket when they need to make clinical decisions. On the same breath, there is a fraction of students who have been reported to misuse technology at the clinical facilities. In their study, Zarandona et al. (2019:69) found that about 23% of participants admitted to having used technology for their own personal gain whilst at the clinical services, whereas 98% admitted that they begin with accessing their social media prior to university resources. Consequently, some nurses at the clinical facilities for WIL perceive technology use by nursing students at the clinical services as unprofessional (Willemse et al. 2019:72).

The above perception by nurses at WIL facilities is different from that of nurse educators at institutions of higher learning or simulation lab. There seems to be an increasing interest by nurse educators and students to adopt technology use in teaching and learning in simulation lab (Foronda et al. 2016:131; Van Vuuren et al. 2018:16). Virtual online learning platforms such as virtual reality have been incorporated into simulation lab to provide immersive learning experience for nursing students (Chang & Lai 2021:5). A study by Van Vuuren et al. (2018:15) demonstrated that the use of high-fidelity simulators contributes positively to the reduction of errors in nursing care, thus improving teaching and learning and prioritising patient safety. The use of technology in simulation lab was further reported to be user-friendly by students (Foronda et al. 2016:131). However, successful implementation of technology use in nursing education heavily relies on cooperation by all stakeholders involved, such as institutional management, willingness by educators and willingness by students (Verkuyl & Mastrilli 2017:45).

### Theme 3: Antecedents for technology use in nursing education

For technology use in teaching and learning to be effective, there are several antecedents that need to be in place. In this theme, the antecedents or enablers of technology for teaching and learning are discussed.

#### Antecedents for technology use in classroom nursing education

As much as there are still challenges to adapt to technology use in nursing education, especially given the current COVID-19 pandemic, it remains a reality that nursing education is shifting to online rather than face-to-face platform. There are several enablers that need to be in place to achieve the goal of technology use in nursing education. Internet facilities, computer hardware and software, students and educator competence in computer and Internet usage are basic antecedents for the use of technology in teaching and learning (Singh et al. 2021:2). On the same breath, senior management and ICT department support is equally an important antecedent for technology use in nursing education (Mackay et al. 2017:03). This is supported by Coopasami et al. (2017:304) who found that psychological readiness, technological readiness and equipment readiness play a critical role as enablers of technology use in nursing education. Therefore, it can be summed up that simply owning a smartphone or a computer with access to Internet does not imply that one is ready for technology use for teaching and learning; readiness goes beyond equipment readiness as it also includes psychological and technological readiness.

### Theme 4: Advantages of technology use in nursing education

There are several studies that have reported the advantages of technology usage in nursing education (Barisone et al. 2019:59; Mackay et al. 2017:3; Toothaker 2018:82). This theme explores the advantages of technology use in nursing education concurrently with its benefits.

#### Advantages of technology use in classroom nursing education

According to Coopasami et al. (2017:305), the use of technology in nursing education is one of the facilitators of self-directed and life-long learning, which are amongst the critical cross-field outcomes. In addition, in a study conducted by Maboe (2017:225), about 40% of the respondents agreed that online discussion forums allowed them to study with their peers, whereas 20% of the respondents reported receiving no support from facilitators when engaged in online learning. This can be substantiated by the fact that when using technology for teaching and learning, tasks are usually completed at their own time and pace and that facilitates learner independence. As Uprichard (2020:272) and Coopasami et al. (2017:305) suggest, the one clear benefit of technology usage in teaching and learning is its flexibility of the location and time when completing tasks. This implies that teaching and learning can happen at any time when either party is at the comfortable location because it is neither time nor space bound.

#### Advantages of technology use in clinical nursing education

In clinical nursing education, the use of technology has risen recently at an alarming rate (O’Connor & Andrews 2018:174). The use of applications, such as virtual reality and virtual patients, is perceived to be convenient, speed up the skills learning process and create a stress-free learning environment (Chang & Lai 2021:5). However, at times, nurse educators find it hard to get relevant audio virtual materials to support learning of such clinical skills (Barisone et al. 2019:58). Thus, equipping nurses with technological skill through the use of technology in clinical nursing education can go a long way in improving the marketability of nursing. This is vital as many nursing education institutions and health establishments are slowly going green and require technological skills in their potential incumbents.

### Theme 5: Disadvantages of technology usage in nursing education

This theme discusses the disadvantages of technology use in both clinical and classroom nursing education. The disadvantages are discussed concurrently with the challenges.

#### Disadvantages and challenges of technology use in classroom nursing education

The shift to virtual approach of teaching and learning from traditional teaching overnight amidst COVID-19 lockdown regulations came as a huge challenge for many educational institutions to adapt to such sudden change. Network-related issues which include audio virtual disparities, interruption of sessions because of unexpected logging out from network and continuous buffering are amongst the challenges experienced when using technology for teaching and learning (Sigh et al. 2021:2). Moreover, poor connectivity and technological illiteracy are also the challenges related to the use of technology for teaching and learning in nursing education (Suliman et al. 2021:3; Uprichard 2020:273). In addition, participants reported lack of time management between family responsibilities and online learning amongst married couples, which was seen as a disadvantage of technology use in classroom nursing education (Suliman et al. 2021:4).

#### Disadvantages and challenges of technology use in clinical nursing education

As much as the use of technology in nursing education should not lose the unique potentiality of nurse–person relationship, it seems to be lacking human interaction (Uprichard 2020:273). This is seen as a disadvantage when using technology for clinical nursing education, in a sense that, in the nursing profession, nurse–patient interaction and relation is vital as it plays a role in facilitating the tridomains of competence, namely, psychomotor, affect and cognitive. As a result, some clinical staff perceive the use of technology devices negatively; it is seen more as a social than an educational tool (Mackay et al. 2017:3).

### Theme 6: Technology use in nursing education amidst COVID-19

It is almost impossible to divorce technology use in nursing education with COVID-19 pandemic, especially in this era. This pandemic has indeed changed the teaching–learning landscape by navigating it to an abrupt online modality of teaching and learning, thus making technology use in nursing education unpopular and unfamiliar to nursing students because of a lack of preparation for its introduction (Oducado & Soriano 2021:8). Although the main purpose for the shift to fully use technology for teaching and learning amidst the COVID-19 pandemic was mainly to safe the academic year, several researchers reported mainly on the challenges that were brought about the implementation of this modality of teaching and learning (Mawere et al. 2021:53; Oducado & Soriano 2021:8; Singh et al. 2021:6). Such challenges included (1) the lack of training for both students and educators on technology-supported teaching and learning, (2) the lack of infrastructure that enables technology-supported teaching and learning and (3) the lack of devices that are necessary for technology-supported teaching and learning. The challenges were mainly owing to its abrupt introduction and the disparities that exist between the ‘haves’ and the ‘have-nots’. As Singh et al. (2021:6) and Mawere et al. (2021:53) put it, infrastructure for technology use for teaching and learning, including lack of capacity to use technology devices, is a huge challenge facing rural education institutions and disadvantaged students. Nevertheless, the upward trend of technology use remains in the shores of nursing education.

## Discussion

This integrative review offered a contemporarily updated evidence on technology usage for teaching and learning in nursing education. The findings of this integrative review indicated that there is a general adoption of technology usage for teaching and learning in higher learning institutions, although some challenges with regard to full adoption are still noted. This is evidenced by the recent upward trend in technology use for teaching and learning, especially given the current COVID-19 social distancing regulations. A study conducted by O’Connor and Andrews (2018:174) concurs with the results of this review wherein they found that there is a notable upward trend in technology use for teaching and learning. Of the same importance is that this current review summarised evidence of the uses of technology in teaching and learning and its advantages.

The findings of this study further highlight that technology use in nursing education is not limited to classroom boundaries, but goes as far as clinical nursing education as well. Although their uses are different in those two components of nursing education, their aim is to corroborate or to complement each other. However, the acceptance of its use especially by nurses at the clinical facilities for WIL seems to be an issue till date. According to Willemse et al. (2019:72), technology use at clinical facilities is perceived to be unprofessional. Zarandona et al. (2019:69) stated that some students have been reported to be using technology devices for personal issues at least once when at practicals. However, there is no evidence supporting the connection between the negative perception of technology use by nurses and the seldom incorrect use of technology by student nurses.

Like any other approach in teaching and learning, there are disadvantages of technology use in teaching and learning. In this review, the disadvantages are discussed together with the challenges. Poor connectivity and interrupted sessions are amongst the challenges of technology use highlighted by a number of researchers (Maboe 2017:226; Suliman et al. 2021:3; Uprichard 2020:273). Similarly, connectivity which leads to interrupted sessions seems to be an issue in the geographical context of the authors of this current review. In addition, the findings of this integrative review highlighted that some institutions have no capacity to offer technology-based nursing education because of lack of resources, which led to course extension for students. However, the common factor in the articles included in this review is that they were all conducted in urban areas with university students and educators as the study population.

## Limitations of the study

The researchers used ‘technology use’ and ‘nursing education’ as search terms in this study, and there are other surrogate terms to these two terms used to search for literature. As a result, there is a likelihood that if a similar study can be conducted using surrogate terms like ‘health science education for nursing education’ and ‘e-learning for technology use’, it might yield different results. Secondly, the time frame in this study was set to the recent years (2016–2021), which can be a limitation also, given the fact that technology use in general has long been a burning issue given the fast approaching of Fourth Industrial Revolution. Lastly, the researchers focused on a discipline of nursing education in which if the context could be changed, it might yield different results.

## Conclusion

The results of this integrative review showed that despite the few challenges and disadvantages reported with regard to technology use, its use continues to grow in an exponential way. Furthermore, the results showed that technology in nursing education is used in both clinical and classroom teaching to complement learning. However, there is still a gap in its acceptance despite its upward trend. To meet the demands of the Fourth Industrial Revolution and the upward trend of technology use amidst COVID-19 pandemic and possibly beyond, the authors of this study recommend that further studies should explore the acceptance of technology use by educators and students in nursing education. Also, further research is recommended on students’ and educators’ attitude towards technology use for teaching and learning in nursing education.
